# Prevalence and impact of fibrinolytic dysregulation in patients with acute coronary syndromes

**DOI:** 10.1186/s12959-021-00288-5

**Published:** 2021-05-22

**Authors:** Huaibin Wan, Xin Fan, Zhihao Wu, Zhenbang Lie, Daqiang Li, Shaohui Su

**Affiliations:** Department of Cardiology, Dongguan People’s Hospital, Southern Medical University, Dongguan, 523059 Guangdong China

**Keywords:** Fibrinolysis, Thrombelastography, Acute coronary syndrome, Retrospective study

## Abstract

**Objective:**

Dual antiplatelet therapy can reduce coronary thrombosis and improve the prognosis in patients with acute coronary syndrome (ACS). However, there was limited prognostic information about fibrinolytic dysregulation in patients with ACS. This study is aimed to evaluated the prevalence and impact of fibrinolytic dysregulation in patients with acute coronary syndrome (ACS).

**Methods:**

We retrospectively analyzed coagulation and fibrinolysis related indexes of ACS in hospitalized adults with rapid thrombelastography between May 2016 and December 2018. All of the follow-up visits were ended by December 2019. The primary outcome was the occurrence of major adverse cardiovascular events (MACEs), which included unstable angina pectoris, non-fatal myocardial infarction, non-fatal cerebral infarction, heart failure and all-cause death.

**Results:**

Three hundred thirty-eight patients were finally included with an average age of 62.5 ± 12.8 years old, 273 (80.5%) were males, 137(40.5%) patients were with ST-elevation myocardial infraction. Fibrinolysis shutdown (LY30<0.8%) and hyperfibrinolysis (LY30 >3.0%) were observed among 163 (48.2%) and 76(22.5%) patients, respectively. During a total of 603.2 person·years of follow-up period, **77** MACEs occurred (22.8%). Multivariate Cox regression analysis indicated that LY30 [HR: 1.101, 95% CI: 1.010–1.200, *P* = 0.028] was independently correlated with the occurrence of MACEs. The hazard ratios pertaining to MACEs in patients with fibrinolysis shutdown and hyperfibrinolysis compared with those in the physiologic range (LY30: 0.8–3.0%) were 1.196 [HR: 1.196, 95% CI: 0.679–2.109,*P* = 0.535] and 2.275 [HR: 2.275, 95% CI: 1.241–4.172, *P* = 0.003], respectively.

**Conclusions:**

Fibrinolytic dysregulation is very common in selected patients with ACS, and hyperfibrinolysis (LY30 > 3%) is associated with poor outcomes in patients with ACS.

## Introduction

Acute coronary syndrome (ACS), a critical disease in cardiovascular system with high morbidity and mortality, is a kind of spontaneous in situ thrombotic disease in atherosclerotic coronary arteries [1]. Therefore, antithrombotic therapy has become a key strategy of the secondary prevention of coronary heart diseases (CHDs). Evidence-based studies have confirmed that dual antiplatelet therapy based on aspirin and P2Y12 receptor antagonists can reduce coronary thrombosis and improve the prognosis of patients with ACS [2].

.Thromboelastography (TEG) is a commonly utilized test to evaluate the severely injured trauma patients [3, 4], which can continuously monitor the whole process of clotting, including the activation of platelet, coagulation and fibrinolysis of the dynamic change. Admission rapid TEG data can predict in-hospital thromboembolic events [5] and guide volume resuscitation [6], and provide more useful and cost-effective evaluation of the coagulation system than multiple conventional coagulation tests [3, 7]. Notably, either TEG detected hyperfibrinolysis or fibrinolysis shutdown was related to poor prognosis in patients with trauma or severe diseases [8–10]. In patients with CHDs, modified TEG was applied to measure platelet reactivity [11, 12] and coagulation function [13]. However, little is known about abnormal fibrinolysis in patients with CHDs. In this study, we observed the prevalence profiles of abnormal fibrinolysis and tested the hypothesis that fibrinolytic dysregulation could predict clinical outcomes among patients with ACS.

## Methods

### Study population

This was a retrospective analysis of ACS in adults presenting to Dongguan people’s hospital with rapid TEG. We retrieved the medical electronic medical records (EMR) of total 12,754 inpatients between May 2016 and December 2018. ACS included either acute ST-elevation myocardial infarction (STEMI) or non-ST-elevation ACS (NSTEACS) and was defined according to present guidelines [1, 10]. The inclusion criteria were: aged 18 years or older, males or females; patients with ACS receiving a loading dose of aspirin 300 mg and clopidogrel 300 mg, and then 100 mg and 75 mg daily respectively; received a TEG test before coronary revascularization procedure; provided a written informed consent. The exclusive criteria were: patients who took aspirin and clopidogrel irregularly or were intolerable to aspirin or clopidogrel; physical trauma within 3 months; serious infection; liver and/or coagulation dysfunction, and primary or acquired thrombocytopenia. Two senior cardiovascular specialists independently reviewed the correctness of the diagnosis according to the above criteria, and the disputed results were jointly discussed and decided by the members of the research team. The protocol was approved by the ethics committee of Dongguan people’s hospital before conducting.

### Data collection

Data collection included the patient’s gender, age, risk factors or medical history, such as the history of smoking, hypertension, diabetes mellitus, cerebral infarction, chronic kidney diseases (CKDs), atrial fibrillation, heart failure and details of medication. Blood pressure, heart rate, height and weight were measured. The blood routine test (Advia2120, Siemens, Germany), alanine aminotransferase (ALT), serum creatinine and total cholesterol (Backman, USA), activated partial thromboplastin time (APTT), prothrombin time (PT), D-dimers and fibrinogen (STAGO STAR, France) were tested according to the standardized operation procedure of our hospital during the first visit.

### Thromboelastography

The fasting antecubital venous whole blood samples was collected by 1:9 sodium citrate tubes (Jingz, Nanchang, China) from patients with ACS at least 6 h after taking loading doses of clopidogrel 300 mg and aspirin 300 mg. The samples were processed by TEG5000 coagulation analyzer (Haemonetics management Co., Ltd., Shanghai, China) according to manufactory’s instruction, and tested within 2 h of collection. All laboratory tests were processed by our hospital’s central laboratory. The following TEG parameters were recorded: R time (time to clot initiation), K-time (total clotting time), angle (the slope between R and K, represents clot kinetics), maximal amplitude (MA, maximal strength of the clot), LY30 (percent clot lysis at 30 min after MA), adenosine diphosphate inhibition rate (ADP-IR) and arachidonic acid inhibition rate (AA-IR). LY30 reflects the rate of fibrinolysis. Based on the previous studies [9, 11, 14], patients were categorized according to their fibrinolytic phenotypes as determined by their LY30 as follows: hyperfibrinolysis was defined as LY30 > 3%, fibrinolysis shutdown as LY30 of less than 0.8% and physiologic fibrinolysis as LY30 between 0.8 and 3%.

### Follow-up and outcomes

We firstly retrieved the follow-up information from our outpatient, emergency and rehospitalization records in the EMR system. If a corresponding visit record could not be obtained, researchers would perform a telephone interview. All of the follow-up visits were ended by December 2019. The primary outcome was major adverse cardiovascular events (MACEs), which included unstable angina pectoris, non-fatal myocardial infarction, non-fatal cerebral infarction, heart failure and all-cause death. Also, other leading to hospitalized adverse events, such as major bleeding, which was defined as bleeding associated with a reduction in hemoglobin of more than 20 g/L or leading to a transfusion of more than 2 units of blood or packed cells or symptomatic bleeding into a critical area or organ, during the follow-up period were recorded.

### Statistical analysis

Continuous variables were reported as mean ± standard deviation if homogeneous, or as median (interquartile range). We used analysis of variance to compare continuous variables among groups. Categorical variables were reported as counts (percentages) and compared with Pearson chi-square test (or fisher exact test). The risk factors of the MACEs were analyzed and carried out by time-to-first event analysis using a Cox regression model. A forward / stepwise regression method was used for multiple factors regression, *P* < 0.05, *P* > 0.10. The correlators of LY30 were analyzed by Pearson bivariate correlation analysis. The risk of MACEs was descripted by the Kaplan-Meier curve. All hypothesis tests were 2-sided and carried out at a significance level of 0.05. Analyses were performed with SPSS 22.0 software package for Windows (SPSS, IBM, USA).

## Results

**Fig. 1:**
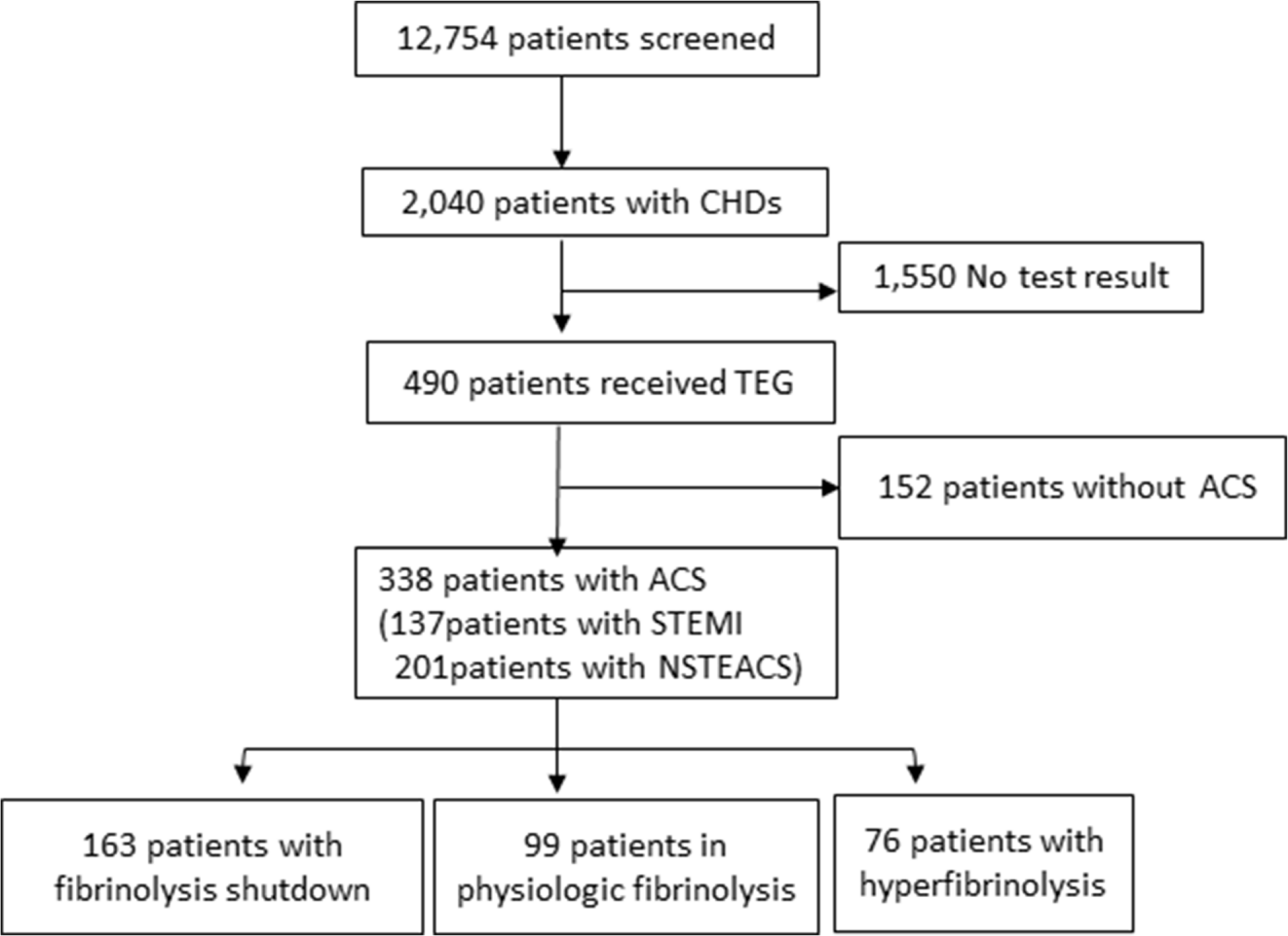
Flow diagram of the study

During the period between May 2016 and December 2018, 490 patients with ACS had at least once TEG test (Fig. 1). According to the inclusion criteria and exclusion criteria, 338 patients with ACS were finally included in the study with an average age of 62.5 ± 12.8 years old, 272 (80.5%) were males, 137(40.5%) patients were with STEMI. According to the result of LY30, fibrinolysis shutdown was the most common phenotype (163 cases, 48.2%), followed by physiologic (99 cases, 29.3%) and hyperfibrinolysis (77cases, 22.5%). There were elevated serum creatinine and total cholesterol, reduced R- and K-time, and increased angle and AA-IR in patients with hyperfibrinolysis. Fibrinogen increased significantly in those patients with fibrinolytic dysregulation (*P* = 0.041). No difference was observed in troponin I, D-dimers, APTT or PT among the groups. Table 1 lists the clinical characteristics of the patients included in this study.

**Table 1:** The clinical characteristics of enrolled patients with acute coronary syndromes

Variable	Total	Fibrinolysis			*P*-value
		Physiologic	Shutdown	Hyper-	
n	338	99	163	76	–
Males, n(%)	272 (80.5)	84 (30.9)	134 (49.3)	54 (19.9)	0.055
Age, years	62.5 ± 12.8	62.6 ± 13.3	62.4 ± 13.1	62.5 ± 11.6	0.457
Smoking, n(%)	199 (58.9)	59 (59.6)	97 (59.5)	43 (56.6)	0.899
Hypertension, n(%)	150 (44.4)	41 (41.4)	70 (42.9)	39 (51.3)	0.373
Diabetes, n(%)	76 (22.5)	28 (28.3)	30 (18.4)	18 (23.7)	0.171
STEMI, n(%)	137 (40.5)	38 (38.4)	69 (42.3)	30 (39.5)	0.801
Stroke, n(%)	12 (3.6)	3 (3.0)	5 (3.1)	4 (5.3)	0.657
CKD, n(%)	22 (6.5)	9 (9.1)	8 (4.9)	5 (6.6)	0.412
CHF, n(%)	11 (3.2)	3 (3.0)	7 (4.3)	1 (1.3)	0.476
SBP, mmHg	134.5 ± 23.9	132.5 ± 25.8	134.3 ± 22.7	137.2 ± 23.9	0.202
Heart rate, beats/min	80.6 ± 16.5	81.0 ± 18.23	80.5 ± 16.1	80.4 ± 15.2	0.254
BMI, kg/m^2^	24.4 ± 3.5	24.0 ± 3.0	24.7 ± 3.9	24.1 ± 3.1	0.228
Troponin I, ng/ml	13.3 (0.94–60.0)	18.5 (1.3–66.8)	13.7 (1.6–74.2)	7.55 (0.33–52.0)	0.229
Serum Creatine, umol/L	87.9 (71.4–112.9)	87.9 (73.1–99.4)	86.3 (70.1–119.5)	90.1 (72.5–129.0)	0.036
ALT, U/L	31.2 (19.3–54.4)	31.2 (20.0–54.7)	31.8 (19.2–54.6)	29 (18.8–50.2)	0.762
Leukocytes, ×10^9^/L	9.8 ± 3.7	9.6 ± 3.7	10.0 ± 3.9	9.4 ± 3.2	0.244
Hemoglobin, g/L	129.0 ± 22.5	130.9 ± 20.2	130.1 ± 23.5	124.2 ± 22.9	0.463
Platelets, ×10^9^/L	217.7 ± 66.6	214.1 ± 69.3	214.9 ± 63.5	228.3 ± 69.1	0.637
Total cholesterol, mmol/L	4.8 ± 1.4	4.7 ± 1.2	4.7 ± 1.3	5.1 ± 1.9	0.026
D-dimers, ug/mL	0.47 (0.28–0.96)	0.48 (0.30–1.22)	0.47 (0.27–0.91)	0.43 (0.30–0.85)	0.790
Fibrinogen, g/L	3.97 ± 1.27	3.79 ± 1.00	4.06 ± 1.46	3.98 ± 1.13	0.041
APTT, sec	39.1 ± 12.8	39.1 ± 11.7	38.8 ± 9.9	39.6 ± 18.6	0.504
PT, sec	13.6 ± 3.4	13.2 ± 1.8	13.9 ± 4.5	13.4 ± 1.7	0.104
R-time, min	5.53 ± 2.79	5.13 ± 2.09	6.04 ± 3.46	4.98 ± 1.45	0.003
K-time, min	1.66 ± 1.00	1.54 ± 0.55	1.86 ± 1.31	1.38 ± 0.46	<0.001
Angle, degree	67.41 ± 8.90	68.32 ± 6.51	65.5 ± 10.7	70.28 ± 5.93	<0.001
MA, mm	64.37 ± 7.99	64.32 ± 6.12	64.67 ± 9.15	63.82 ± 7.51	0.082
LY30, %	0.8 (0–2.6)	1.6 (1.10–2.10)	0 (0–0.20)	5.05 (3.92–7.40)	0.001
ADP-IR, %	47.6 ± 32.8	45.7 ± 32.9	50.8 ± 33.1	43.4 ± 31.7	0.736
AA-IR, %	71.2 ± 28.9	66.6 ± 32.3	70.4 ± 28.3	78.4 ± 24.5	0.009

Notes: *STEMI* ST-elevation myocardial infarction, *CKD* Chronic kidney diseases, *CHF* Congestive heart failure, *SBP* Systolic blood pressure, *BMI* Body mass index = weight (kg)/height(m)2, *ALT* Alanine aminotransferase, *APTT* Activated partial thromboplastin time, *PT* prothrombin time, *MA* Maximal amplitude, *LY30* Lysis 30 min after MA, *ADP-IR* Adenosine diphosphate inhibition rate, *AA-IR* Arachidonic acid inhibition rate

Twenty-one patients (6.2%) were lost to follow-up during the first year. Our median follow-up period was 708 days (interquartile range: 323 to 1037 days). During a total of 603.2 person·years of follow-up period, 77 MACEs occurred (22.8%), including ischemic cardiovascular events 35 (10.4%), heart failure 24 (7.1%), transient ischemic attack (TIA) or non-fatal cerebral infarction 26 (7.7%), and all-cause death 17 (5.0%), respectively. 9(2.7%) bleeding events were observed. Table 2 lists the clinical outcomes in details. There was an increasing trend of MACEs in patients with hyperfibrinolysis (*P* = 0.057). Compare to physiologic state (3%), stroke or TIA was significantly increased in settings of fibrinolysis dysregulation (shutdown 8%, hyperfibrinolysis 13.2%, *P* = 0.044).

**Table 2:** Clinical outcomes during the follow-up period

Events	Total *N* = 338	Fibrinolysis			*P* value
		Physiologic *N* = 99	Shutdown *N* = 163	Hyper- *N* = 76	
MACEs^a^	77 (22.8)	19 (19.2)	33 (20.2)	25 (32.9)	0.057
All-cause death	17 (5.0)	4 (4.0)	10 (6.3)	3 (3.9)	0.668
ACS	35 (10.4)	9 (9.1)	14 (8.6)	12 (15.8)	0.208
Stroke/TIA	26 (7.7)	3 (3.0)	13 (8.0)	10 (13.2)	0.044
Heart failure	24 (7.1)	4 (4.0)	11 (6.7)	9 (11.8)	0.134
Bleeding	9 (2.7)	4 (4.0)	4 (2.5)	1 (1.3)	0.540

Notes: ^a^*ACS* Acute coronary syndrome, *TIA* Transient ischemic attack, *MACEs* Includes all-cause death, ACS Stroke or TIA, and heart failure

Univariate Cox regression analysis showed that the occurrence of MACEs was significantly correlated with age [HR:1.043, 95%CI:1.022–1.063,P<0.001], systolic blood pressure [HR:1.009, 95% CI:1.000–1.019,*P* = 0.045], hemoglobin [HR:0.984, 95% CI:0.974–0.993,*P* = 0.001], serum creatinine [HR:1.002, 95% CI:1.001–1.003, *P* = 0.003], D-dimers [HR:1.147, 95% CI:1.024–1.285, *P* = 0.018],fibrinogen [HR: 1.193, 95% CI:1.029–1.385,*P* = 0.020], LY30[HR:1.117, 95% CI:1.041–1.198,*P* = 0.002], but not with gender, body mass index (BMI), medical histories (such as smoking, hypertension and diabetes), heart rate, troponin I, leukocytes, platelets, total cholesterol, ALT, PT, APTT, R time, K time, angle, MA, ADP-IR and AA-IR. Multivariate Cox regression analysis based on age, gender, levels of systolic blood pressure, hemoglobin, serum creatinine, D-dimers, fibrinogen and LY30 indicated that only LY30 [HR: 1.097, 95% CI: 1.013–1.188, *P* = 0.023] was independently associated with the occurrence of MACEs. The results of multivariate Cox regression analysis were listed in Table 3**.**

**Table 3:** Multivariate Cox regression analysis pertaining to MACEs in patients with acute coronary syndrome

Variable	1 represents	HR(95%CI)	*P* value
Gender	Male	1.638 (0.774–3.465)	0.197
Age	1-year increment	1.023 (0.995–1.052)	0.101
SBP	1 mmHg increment	1.004 (0.994–1.014)	0.456
Hemoglobin	1 g/l increment	0.992 (0.975–1.009)	0.373
Serum Creatine	1umol/l increment	1.000 (0.998–1.003)	0.762
D-dimers	1μg/ml increment	1.073 (0.920–1.250)	0.370
Fibrinogen	1 g/l increment	1.056 (0.852–1.308)	0.620
Troponin I	1 ng/ml increment	1.001 (0.998–1.004)	0.506
LY30	1% increment	1.101 (1.010–1.200)	0.028

Notes:*ACS* Acute coronary syndrome, *HR* Hazard ratio, *CI* Confidence interval, *SBP* systolic blood pressure, *LY30* Percent clot lysis at 30 min after maximal amplitude. MACEs include all-cause death, ACS, stroke or TIA, and heart failure

**Fig. 2:**
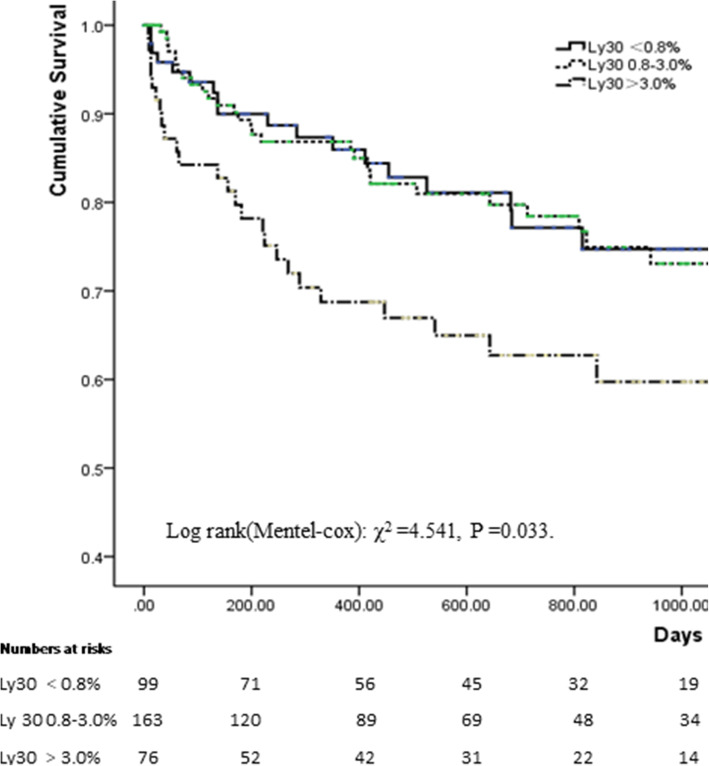
Kaplan-Meier curve

As shown in the Kaplan-Meier curve (see Fig. 2), the levels of LY30>3.0% significantly increased the risk of MACEs (Log Rank (Mentel-cox): χ^2^ = 4.541, *P* = 0.033). When taken LY30 as a categorical variable, multivariate Cox regression analysis indicated that the hazard ratios pertaining to MACEs in patients with LY30<0.8% and >3.0% compared with those in physiologic range (LY30 0.8–3.0%) were 1.196 [HR: 1.196, 95% CI: 0.679–2.109, *P* = 0.535] and 2.275 [HR: 2.275, 95% CI: 1.241–4.172, *P* = 0.003], respectively. Pearson correlation analysis indicated that LY30 was weakly correlated with the level of AA-IR(r = − 0.115, *p* = 0.042), but not with platelet count, ADP-IR, PT, APTT, fibrinogen and D-dimer(details as shown in Table 4).

**Table 4:** Pearson correlation between LY30 and other associated factors

Variable	r	*P* value
Platelet count	0.083	0.129
ADP-IR	−0.077	0.172
AA-IR	−0.115	0.042
PT	−0.053	0.341
APTT	−0.030	0.588
Fibrinogen	−0.020	0.717
D-dimers	−0.041	0.471

Notes: *LY3*0 Lysis 30 min after maximal amplitude, *ADP-IR* Adenosine diphosphate inhibition rate, *AA-IR* Arachidonic acid inhibition rate, *APTT* Activated partial thromboplastin time, *PT* Prothrombin time

## Discussion

Our study observed that more than two thirds of the selected patients with ACS endured abnormal fibrinolysis, which increased the risk of stroke or TIA. Fibrinolysis shutdown was the most common phenotype. However, it is not fibrinolysis shutdown but hyperfibrinolysis (HF) (LY30 > 3%) associated with the occurrence of MACEs in patients with ACS.

The profiles of fibrinolytic dysregulation in patients with ACS were similar as those detected in severely injured patients [8]. In patients with trauma, both fibrinolysis shutdown and hyperfibrinolysis suggest poor prognosis, which indicated a U-shaped association with LY30 and mortality [3, 4]. Our study did not exhibit the association between fibrinolysis shutdown and clinical outcomes. Fibrinolysis shutdown represented a hypercoagulable state in previous studies [8], and early antithrombotic therapy might reduce microvascular thrombi and end-organ injury, as well as thromboembolic events. Thanks to early active antithrombotic strategy, fibrinolysis shutdown did not increase the risk of MACEs in patients with ACS.

D-dimer is a specific byproduct of the enzymatic cleavage of fibrin and its elevation strongly suggests increased fibrinolysis. In our univariate analysis, both D-dimers and fibrinogen were associated with poor prognosis, but the associations were not significant in multivariate analysis after adjusted by LY30 and other variables. In our correlation analysis, LY30 was only slightly correlated with AA-IR, but not correlated with D-dimers, fibrinogen and other parameters. It was possible that LY30 and D-dimers represented different phases of fibrinolysis. D-dimers indicated that fibrinolysis has occurred in vivo. However, the profile of LY30 is based on the power of fibrinolysis in vitro during TEG test, which may reflect a trend or pre fibrinolysis state of whole blood. Therefore, LY30 might be an earlier and more valuable index than D-dimers and other parameters in evaluating the association between fibrinolysis and prognosis.

Our study did not find a relationship between TEG detected platelet inhibition rates and patient’s prognosis, although previous platelet mapping assay via modified TEG showed a good correlation with the turbidimetric light transmittance aggregometry, which considered to be the “gold standard” in assessing platelet function [15]. Moreover, small sample study indicated that TEG directed antiplatelet could improve prognosis [14]. However, a randomized study had indicated that TEG directed dual antiplatelet was not benefit for keeping grafts patency and reducing thromboembolic events in patients undergoing coronary artery bypass surgery [16]. Therefore, the potential for clinical applications of TEG in assessing risk of recurrent ischemic events among patients receiving antithrombotic agents remains unclear, but warrants further investigation.

### Limitations

There are several inherent limitations to this study. First, among 2040 patients with CHDs, only 490 of them had TEG test. The patients who receive a TEG test might be more vulnerable to fibrinolytic dysregulation, so there was a selective bias. Second, this retrospective study focused on the process of coagulation and fibrinolysis, but some long-term drug treatment factors, such as β blockers, ACEIs, ARBs and statins, were not included, which were commonly used in patients with ACS. Third, coagulation, fibrinolysis and bleeding were closely related, only once TEG test could not exhibit the dynamic process, nor to evaluate the benefits of adjusted TEG parameters on clinical outcomes. Moreover, plasma levels of plasmin inhibitor, plasmin-plasmin inhibitor complex and plasminogen activator inhibitor, which may be helpful for corroborating fibrinolysis, had not been routine tested in our clinical laboratory. In addition, due to a limited sample, it is not enough power to assessing the risk of bleeding. Therefore, further studies were needed to answer these unsolved questions.

## Conclusions

Fibrinolysis shutdown was the most common phenotype and likely represents a coagulopathic state in patients with ACS, early antithrombotic therapy might benefit to improve their prognosis. However, hyperfibrinolysis (LY30 > 3%) is still an independent indicator of poor outcomes in patients with ACS, and appropriate clinical interventions still need to explore.

## Data Availability

The datasets are available from the corresponding author on reasonable request.

## Abbreviations

AA-IR: Arachidonic acid inhibition rate
ACS: Acute coronary syndrome;
ADP-IR: Adenosine diphosphate inhibition rate
ALT: Alanine aminotransferase
APTT: Activated partial thromboplastin time
BMI: Body mass index
CHD: Coronary heart diseases
CI: Confidence interval
CKD: Chronic kidney diseases
EMR: Electronic medical records
HF: Hyperfibrinolysis
HR: Hazard ratio
LY30: Lysis 30 min after maximal amplitude
MA: Maximal amplitude
MACE: Major adverse cardiovascular events
NSTEACS: Non ST-elevation ACS
PT: Prothrombin time
STEMI: ST-elevation myocardial infarction
TEG: Thromboelastography
TIA: Transient ischemic attack.
